# Preoperative Serum C-Reactive Protein Levels Are Elevated in Uterine Sarcoma Compared with Leiomyoma: A Retrospective Cohort Study

**DOI:** 10.3390/cancers18071154

**Published:** 2026-04-03

**Authors:** Monika Colja, Marija Batkoska, Luka Kovač, Kristina Drusany Starič

**Affiliations:** 1 Department of Gynecologic Oncology, Institute of Oncology Ljubljana, 1000 Ljubljana, Slovenia; mcolja@onko-i.si; 2 Division of Gynaecology and Obstetrics, University Medical Centre Ljubljana, 1000 Ljubljana, Slovenia; luka.kovac@kclj.si (L.K.); kristina.drusany@kclj.si (K.D.S.); 3 Faculty of Medicine, University of Ljubljana, 1000 Ljubljana, Slovenia

**Keywords:** uterine sarcoma, leiomyoma, C-reactive protein, inflammatory biomarker, oncologic risk stratification, uterine malignancy, tumour-associated inflammation, morcellation risk

## Abstract

Background: Uterine sarcomas are rare but highly aggressive mesenchymal malignancies associated with poor survival. Preoperative differentiation from benign leiomyomas remains a critical oncologic challenge, frequently resulting in unexpected postoperative diagnoses and potential tumour dissemination during morcellation. Reliable, accessible biomarkers to support preoperative risk assessment are lacking. This study evaluated whether CRP, a systemic inflammatory marker implicated in tumour biology, could aid in the preoperative identification of uterine sarcoma. Methods: This retrospective single-centre study included 39 patients with histologically confirmed uterine sarcoma and 39 patients with leiomyoma treated between 2010 and 2021. Preoperative serum CRP levels were compared between groups. As data were non-normally distributed, the Mann–Whitney U test was used for comparisons, and Spearman’s rank correlation was applied for association analyses. Results: Patients with sarcoma were significantly older than controls (56.2 ± 12.9 vs. 39.2 ± 6.7 years, *p* < 0.0001). Preoperative CRP levels were significantly higher in sarcoma patients compared with leiomyoma patients (26.4 ± 46.8 mg/L vs. 0.4 ± 1.6 mg/L; *p* < 0.001). Elevated CRP (>5 mg/L) was observed in 53.8% of sarcoma cases versus 2.6% of controls. Undifferentiated sarcomas demonstrated the highest CRP levels. CRP levels were not significantly associated with tumour aggressiveness. A moderate negative correlation between age and preoperative CRP was identified (r = −0.476, *p* = 0.029). Receiver operating characteristic analysis demonstrated a moderate discriminatory ability of preoperative CRP for differentiating uterine sarcoma from leiomyoma (AUC 0.751, 95% CI 0.668–0.834). Conclusions: Elevated preoperative CRP levels are significantly associated with uterine sarcoma and may enhance oncologic risk stratification prior to surgery. Integration of CRP into multimodal preoperative assessment algorithms could improve surgical planning and reduce the risk of inadvertent tumour dissemination. Prospective multicentre studies are required to validate its diagnostic performance and define clinically relevant cut-off values.

## Simple Summary

Uterine sarcomas are rare and aggressive tumours that are often difficult to distinguish from benign uterine fibroids before surgery. Accurate preoperative diagnosis is crucial to avoid inappropriate surgical techniques such as morcellation, which may worsen prognosis if malignancy is present. In this retrospective study, we evaluated whether serum C-reactive protein (CRP), an inexpensive and widely available inflammatory marker, could help differentiate uterine sarcomas from leiomyomas. We found significantly higher preoperative CRP levels in patients with sarcoma than in those with leiomyoma. These findings suggest that CRP may serve as an accessible adjunct marker in the preoperative assessment of suspected uterine malignancy.

## 1. Introduction

Uterine sarcomas are rare but highly aggressive mesenchymal malignancies, accounting for approximately 1% of all gynecological cancers and 3–7% of uterine malignancies, with an annual incidence of 1.55–1.95 per 100,000 women [1]. Compared with epithelial uterine cancers, they exhibit more aggressive biological behaviour and poorer survival outcomes [2,3]. Histologically, uterine sarcomas include leiomyosarcomas and endometrial stromal sarcomas (ESS), which are further divided into low- and high-grade subtypes, undifferentiated sarcomas, and adenosarcomas [4,5]. Tumour stage remains the most important prognostic factor, with 5-year survival rates ranging from 50 to 70% in stage I disease to below 20% in advanced stages [3].

Established risk factors include postmenopausal status, Black ethnicity, prolonged tamoxifen use, and prior pelvic irradiation [1,6,7,8]. Incidence increases with age, reaching 6.4 per 100,000 women over 50 years [1]. Despite advances in imaging, reliable preoperative differentiation between uterine sarcoma and benign leiomyoma remains a major clinical challenge. Clinical presentation is nonspecific and frequently overlaps with that of leiomyomas, which affect up to 70% of women [9,10]. Consequently, sarcomas are detected retrospectively in 0.1–0.3% of surgeries performed for presumed benign fibroids [11,12,13]. This diagnostic uncertainty carries important oncologic implications, particularly regarding surgical planning and the potential risk of tumour dissemination during morcellation.

Chronic inflammation is increasingly recognized as a hallmark of cancer, contributing to tumour initiation, progression, angiogenesis, and immune modulation [14,15,16,17]. C-reactive protein (CRP), an acute-phase reactant synthesized in response to proinflammatory cytokines, has demonstrated prognostic value in several malignancies, including ovarian and endometrial cancer, as well as in soft-tissue sarcomas [15,18,19,20,21,22,23,24,25]. Elevated CRP levels have been associated with worse survival and more aggressive tumour behaviour in these settings. However, while CRP has been investigated as a prognostic marker in established uterine leiomyosarcoma [22], its potential role as a preoperative diagnostic biomarker to distinguish uterine sarcoma from benign leiomyoma has not been systematically evaluated.

Given the persistent difficulty in preoperative risk stratification and the absence of reliable serum biomarkers for uterine sarcoma, there remains a clear unmet clinical need for accessible adjunct diagnostic tools. The present study investigates whether preoperative serum CRP levels may support the differentiation of uterine sarcoma from leiomyoma and thereby improve oncologic decision-making prior to surgery.

Despite advances in imaging modalities such as ultrasound and magnetic resonance imaging (MRI), no single technique has demonstrated sufficient sensitivity and specificity for reliable preoperative diagnosis of uterine sarcoma. MRI features such as irregular margins, heterogeneous signal intensity, and areas of necrosis may raise suspicion; however, significant overlap with atypical leiomyomas limits diagnostic accuracy [22,26,27]. Similarly, Doppler ultrasound findings, including increased vascularity, are not specific for malignancy. Consequently, current diagnostic approaches rely on a combination of clinical, radiological, and intraoperative findings, none of which are definitive. This diagnostic uncertainty underscores the need for adjunctive biomarkers that are inexpensive, widely available, and easily integrated into routine preoperative assessment.

## 2. Materials and Methods

### 2.1. Study Design and Patients

This retrospective single-centre study included all patients with histologically confirmed uterine sarcoma who underwent surgical treatment between January 2010 and December 2021 at the Department of Gynecology, University Medical Centre Ljubljana, Slovenia. The surgical approach (laparotomy vs. minimally invasive surgery) was recorded from the medical records as part of the clinical dataset. However, no formal analysis of the association between operative approach and CRP levels was undertaken in the present study.

In an exploratory subgroup analysis, uterine sarcomas were classified as aggressive or non-aggressive according to histologic subtype. Leiomyosarcoma, high-grade endometrial stromal sarcoma, and undifferentiated uterine sarcoma were categorized as aggressive tumours, whereas low-grade endometrial stromal sarcoma and adenosarcoma were categorized as non-aggressive tumours, based on their established biological behaviour and clinical course.

The control group consisted of 39 consecutive patients who underwent surgery during the same period for histologically confirmed uterine leiomyoma. The availability of preoperative serum CRP values was required for inclusion. Clinical, laboratory, and histopathological data were retrieved from institutional electronic medical records.

**Figure 1:**
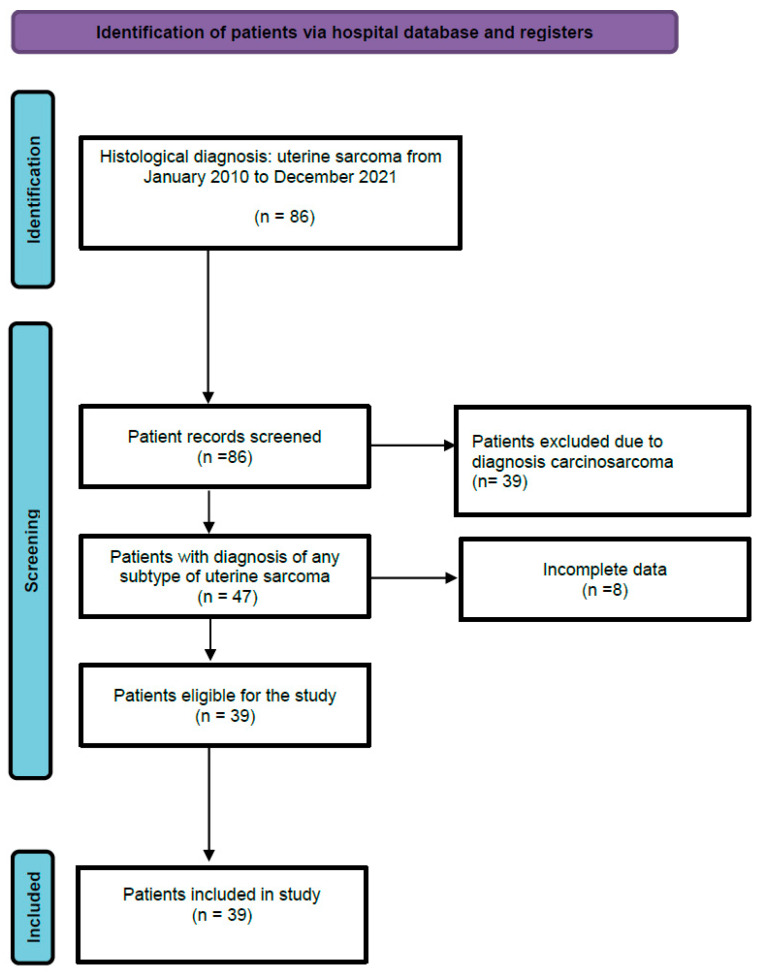
Flowchart of patient selection. The diagram shows the stepwise inclusion process of patients with histologically confirmed uterine sarcoma between January 2010 and December 2021. Patients with carcinosarcoma or incomplete data were excluded. The final cohort included 39 eligible cases.

Exclusion criteria included histological diagnosis of carcinosarcoma, incomplete clinical data, documented acute infection at the time of CRP measurement, or other clinically evident inflammatory conditions. The patient selection process is summarized in Figure 1.

### 2.2. CRP Measurement

Serum CRP levels were measured routinely as part of standard preoperative laboratory evaluation. CRP concentrations were determined using a standardized immunoturbidimetric assay in the central hospital laboratory and reported in mg/L. Values > 5 mg/L were considered elevated according to institutional reference ranges. Postoperative CRP was measured on postoperative day 1 as part of routine postoperative laboratory assessment.

Leukocyte count was available in routine preoperative laboratory testing but was not included in the present analysis, which focused specifically on CRP. Differential blood count was not routinely assessed preoperatively at our institution and was therefore not available in a standardized form. Although BMI and metabolic comorbidities could be retrieved from the medical records, they were not systematically incorporated into the current analysis. Smoking status was not consistently documented.

### 2.3. Ethical Approval

The study protocol was approved by the National Ethics Committee of the Republic of Slovenia (No. 0120-477/2020/5). The study was conducted in accordance with the Declaration of Helsinki.

### 2.4. Statistical Analysis

Statistical analysis was performed using IBM SPSS Statistics (Version 29.0, IBM Corp., Armonk, NY, USA). All tests were two-sided, and *p* < 0.05 was considered statistically significant. Data distribution was assessed using the Shapiro–Wilk test. Because CRP values were non-normally distributed, between-group comparisons for continuous variables were performed using the Mann–Whitney U test, and correlations were assessed using the Spearman’s rank correlation coefficient. Continuous variables are presented as mean ± standard deviation for descriptive comparability with prior oncologic literature and additionally as median with interquartile range to better reflect the skewed distribution. Categorical variables were compared using the chi-square test or Fisher’s exact test, as appropriate. Descriptive subgroup analyses were performed according to histologic subtype and tumour aggressiveness. Owing to the limited sample size and retrospective design, receiver operating characteristic analysis and cut-off derivation were considered exploratory and were not used as the basis for definitive diagnostic performance conclusions.

## 3. Results

### 3.1. Patient Characteristics

**Figure 2:**
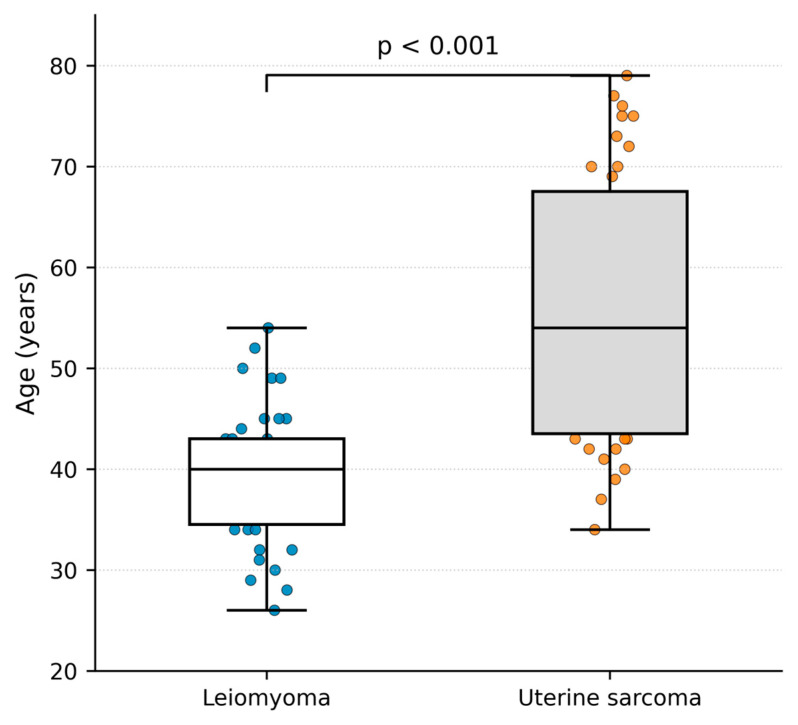
Age distribution in patients with uterine sarcoma and leiomyoma. Box-and-whisker plots with overlaid individual data points show patient age at surgery. The box indicates the interquartile range (IQR), the horizontal line indicates the median, and whiskers extend to 1.5 × IQR.

A total of 78 patients were included in the study: 39 with histologically confirmed uterine sarcoma and 39 with uterine leiomyoma. The mean age of patients with uterine sarcoma was significantly higher than that of patients with leiomyoma (56.2 ± 12.9 vs. 39.2 ± 6.7 years, *p* < 0.0001) (Figure 2).

At the time of diagnosis, 33.3% of sarcoma patients were younger than 50 years, while 66.7% were aged 50 years or older. In contrast, only 2.6% of patients with leiomyoma were aged ≥50 years, whereas 97.4% were younger than 50 years.

The operative approach differed according to preoperative clinical suspicion and tumour characteristics; however, owing to the retrospective design and limited sample size, no adjusted comparison of CRP by surgical approach was performed.

Among the 39 uterine sarcoma cases, leiomyosarcoma was the most common subtype (13/39, 33.3%), followed by adenosarcoma (10/39, 25.6%), low-grade ESS (6/39, 15.4%), high-grade ESS (5/39, 12.8%), and undifferentiated sarcoma (5/39, 12.8%). Among the 21 sarcoma patients with elevated preoperative CRP values (>5 mg/L), the distribution of histologic subtypes was as follows: leiomyosarcoma, 7/21 (33.3%); adenosarcoma, 6/21 (28.6%); undifferentiated sarcoma, 5/21 (23.8%); high-grade ESS, 2/21 (9.5%); and low-grade ESS, 1/21 (4.8%).

### 3.2. Preoperative CRP Levels in Sarcoma and Leiomyoma

Among the 39 patients with uterine leiomyoma, 38 (97.4%) had normal preoperative CRP levels. Elevated CRP values (>5 mg/L) were observed in 1 patient (2.6%), with a mean CRP level of 0.4 ± 1.6 mg/L (Table 1).

Median CRP values followed the same pattern as mean values, supporting the robustness of the observed between-group difference despite the skewed distribution.

In the uterine sarcoma group, elevated preoperative CRP levels were detected in 21 of 39 patients (53.8%), whereas 18 patients (46.2%) had normal CRP values. The mean preoperative CRP level in sarcoma patients was 26.4 ± 46.8 mg/L (Table 2).

**Figure 3:**
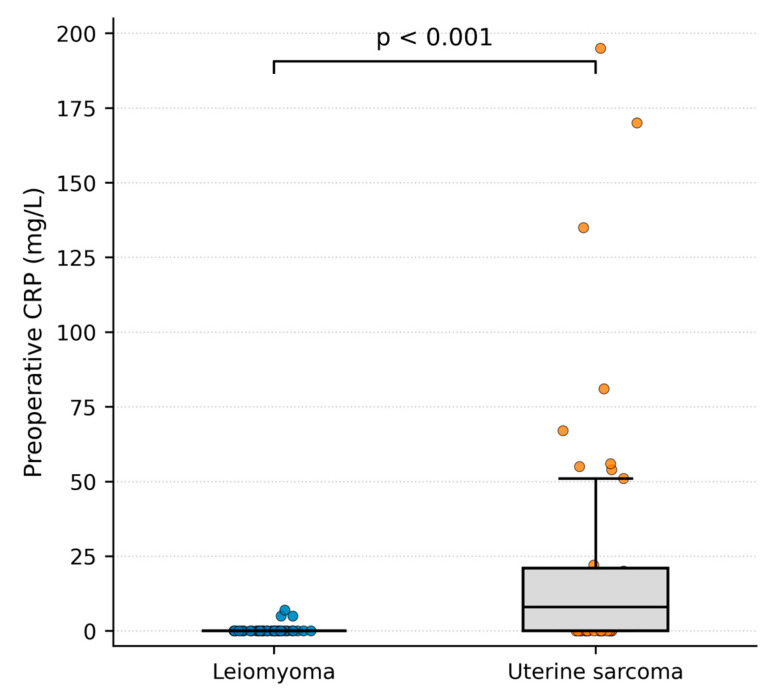
Preoperative CRP levels in patients with uterine sarcoma and leiomyoma (Mann–Whitney U test). Box-and-whisker plots with overlaid individual data points show preoperative serum CRP concentrations in mg/L.

Comparison between groups using the Mann–Whitney U test demonstrated significantly higher preoperative CRP levels in patients with uterine sarcoma compared with those with leiomyoma (*p* < 0.001) (Figure 3).

**Figure 4:**
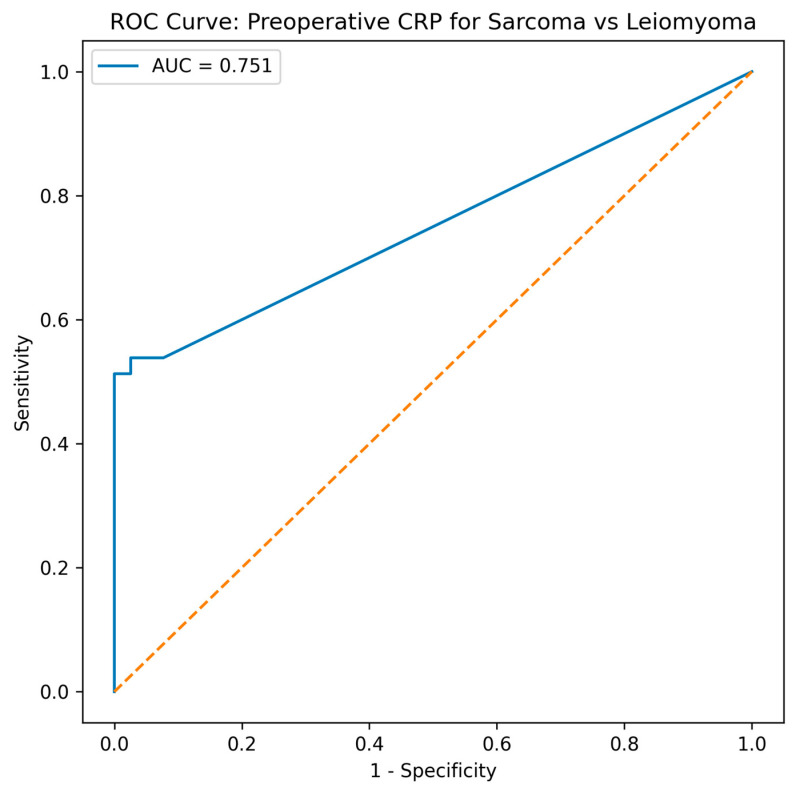
ROC curve of preoperative serum CRP for differentiating uterine sarcoma from leiomyoma. The AUC was 0.751 (95% CI 0.668–0.834), indicating moderate discriminatory ability. The dashed diagonal line indicates chance-level discrimination (AUC = 0.5).

Receiver operating characteristic (ROC) analysis showed that preoperative CRP had moderate discriminatory ability for differentiating uterine sarcoma from leiomyoma, with an area under the curve (AUC) of 0.751 (95% CI 0.668–0.834). Using a threshold of >5 mg/L, sensitivity was 53.8%, specificity 97.4%, positive predictive value 95.5%, negative predictive value 67.9%, and overall accuracy 75.6%. The optimal cut-off in this dataset, according to the Youden index, was approximately >7 mg/L, yielding a sensitivity of 51.3% and specificity of 100.0%. Data are graphically shown in Figure 4.

### 3.3. CRP Levels According to Sarcoma Subtype

**Figure 5:**
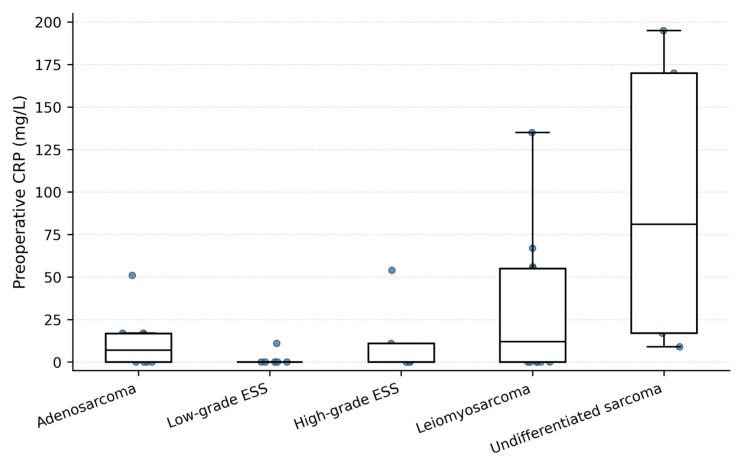
Preoperative CRP levels according to uterine sarcoma subtype. Box-and-whisker plots with overlaid individual data points show preoperative CRP values across histologic subtypes.

CRP levels varied across different histological subtypes of uterine sarcoma (Table 3). The distribution of preoperative CRP values across sarcoma subtypes is shown in Figure 5. Undifferentiated sarcomas demonstrated the highest mean preoperative CRP levels (94.4 ± 50.2 mg/L), followed by high-grade ESS (54.2 ± 40.3 mg/L) and leiomyosarcoma (56.8 ± 41.9 mg/L). Lower CRP levels were observed in low-grade ESS (11.0 ± 8.5 mg/L) and adenosarcoma (17.3 ± 9.6 mg/L).

A similar pattern was observed in postoperative CRP levels, which remained highest in patients with undifferentiated sarcoma (117.6 ± 62.1 mg/L) and lowest in those with low-grade ESS (15.7 ± 10.4 mg/L).

### 3.4. CRP Levels and Tumour Aggressiveness

Statistical analysis using the Mann–Whitney U test revealed no significant difference in preoperative CRP levels between aggressive and non-aggressive sarcoma subtypes (*p* = 0.370) (Table 4).

Similarly, postoperative CRP levels (*p* = 0.214) and changes in CRP levels between preoperative and postoperative measurements (*p* = 0.743) were not significantly associated with tumour aggressiveness.

Spearman correlation analysis also demonstrated no significant association between CRP levels and tumour aggressiveness (preoperative CRP: r = 0.209, *p* = 0.364).

### 3.5. Correlation Between Age and CRP

A significant negative correlation was observed between patient age and preoperative CRP levels (r = −0.476, *p* = 0.029), indicating that younger patients tended to have higher CRP levels.

### 3.6. Postoperative CRP Levels

**Figure 6:**
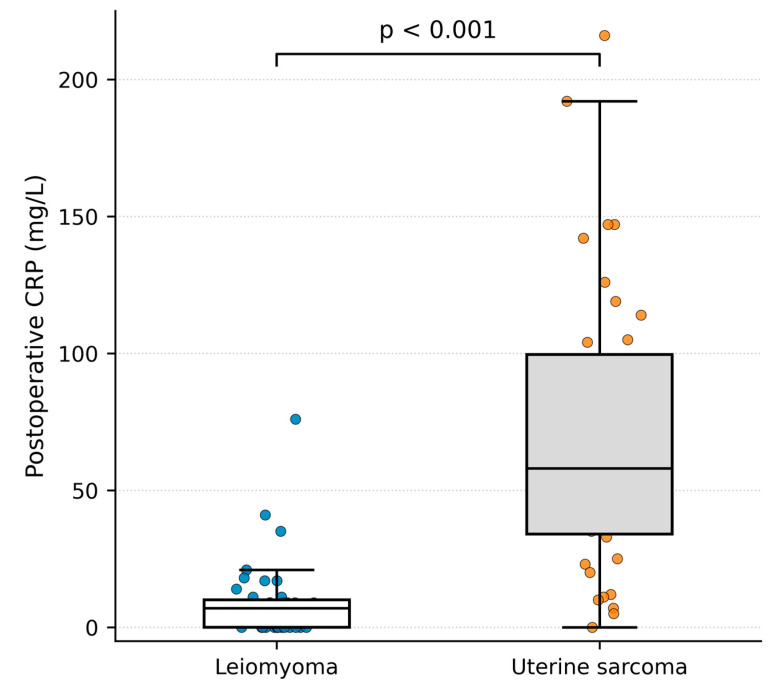
Postoperative CRP levels in patients with uterine sarcoma and leiomyoma. Box-and-whisker plots with overlaid individual data points show postoperative serum CRP concentrations in mg/L.

Postoperative CRP levels were significantly higher in patients with uterine sarcoma compared with those with leiomyoma (*p* < 0.0001) (Figure 6).

## 4. Discussion

The present study demonstrates that preoperative serum CRP levels are significantly higher in patients with uterine sarcoma compared with those diagnosed with benign uterine leiomyoma. These findings suggest that systemic inflammatory markers may contribute to the preoperative assessment of patients presenting with uterine masses. To our knowledge, this is the first study evaluating CRP as a potential biomarker for distinguishing uterine sarcoma from leiomyoma prior to surgery. While patients with leiomyomas consistently exhibited low CRP levels, elevated CRP values were frequently observed in sarcoma patients, supporting the potential role of CRP as an adjunct diagnostic marker in the preoperative evaluation of suspected uterine malignancy.

The association between systemic inflammation and cancer development is well established. Chronic inflammatory processes can promote carcinogenesis through mechanisms including DNA damage, stimulation of angiogenesis and inhibition of apoptosis pathways [16]. Proinflammatory cytokines such as interleukin-1, interleukin-6, tumour necrosis factor-α and transforming growth factor stimulate hepatic CRP production and contribute to tumour growth, survival and migration [17,28,29]. Tumours often arise in inflammatory microenvironments, where cytokine-mediated signalling pathways regulate angiogenesis, tumour progression and immune responses [16]. As an acute-phase reactant produced primarily by hepatocytes, CRP reflects systemic inflammatory activity and has been extensively studied as a prognostic biomarker in several malignancies.

Elevated serum CRP levels have been associated with worse outcomes in multiple cancers, including colorectal [18,19,20,30,31], oesophageal [19,32], gastric [33], hepatocellular [20], and renal carcinomas [21]. In gynaecologic oncology, elevated CRP has also shown prognostic significance in ovarian, cervical and endometrial cancers [22,23,34], supporting the broader relevance of inflammatory biomarkers in female genital tract malignancies. For example, Hefler et al. demonstrated that increased preoperative CRP levels were independently associated with poorer survival in patients with ovarian cancer [15], while Schmid et al. reported similar findings in surgically treated endometrial cancer patients [23]. In cervical adenocarcinoma, pre-treatment CRP levels have also been shown to predict prognosis [24]. These findings support the concept that systemic inflammatory responses may reflect tumour burden or aggressive tumour biology.

Our results are consistent with these observations and extend them to uterine sarcomas. Patients with sarcoma in our cohort exhibited significantly higher CRP levels compared with patients with leiomyoma, suggesting that malignant uterine tumours may induce a more pronounced systemic inflammatory response even prior to surgical intervention. Similar associations between CRP and disease characteristics have been described in soft-tissue sarcomas, where elevated CRP levels have been linked to tumour progression and poorer clinical outcomes [22]. Although our analysis showed variability in CRP levels across different sarcoma subtypes, these differences were not statistically significant, indicating that CRP alone may not reliably distinguish between individual sarcoma histologies.

Interestingly, our study identified a negative correlation between patient age and preoperative CRP levels, with younger patients demonstrating higher CRP concentrations. While CRP levels generally increase with age in the general population, the observed pattern may reflect differences in tumour biology or immune response among patients with uterine sarcoma. However, further investigation is required to clarify the mechanisms underlying this finding. In age-adjusted logistic regression, age remained significantly associated with sarcoma status, whereas preoperative CRP did not retain independent statistical significance, suggesting that CRP should be interpreted as an adjunctive rather than stand-alone marker.

In addition, postoperative CRP levels remained significantly higher in patients with uterine sarcoma compared with those with leiomyoma. This persistent elevation may indicate a stronger inflammatory response associated with malignant disease or a slower resolution of postoperative inflammation in sarcoma patients. Nevertheless, the clinical significance of postoperative CRP dynamics in uterine sarcoma remains unclear and warrants further study.

Although uterine sarcomas are rare, they are characterized by aggressive clinical behaviour, high recurrence rates, and poor overall survival [12]. This is consistent with recent retrospective cohort data describing the unfavourable clinical course of uterine sarcoma [35]. Accurate preoperative differentiation from benign leiomyomas remains challenging, as imaging modalities and clinical presentation often overlap. Consequently, many sarcomas are diagnosed only after surgical removal of a presumed benign tumour. In this context, accessible biomarkers such as CRP may offer additional information that could support preoperative risk assessment and surgical planning.

Importantly, this study addresses a clinically relevant gap, as no validated serum biomarkers are currently available to support preoperative differentiation of uterine sarcoma from leiomyoma. From a clinical perspective, the identification of a simple and accessible biomarker such as CRP could have important implications for surgical decision-making. In cases where uterine malignancy is suspected, surgical strategies differ substantially from those used for benign disease, particularly with regard to the avoidance of power morcellation [36]. Morcellation of an undiagnosed uterine sarcoma has been associated with increased risk of intra-abdominal dissemination and worse oncologic outcomes. Therefore, even modest improvements in preoperative risk stratification may translate into clinically meaningful benefits.

The present findings may also be relevant to the choice of surgical approach. In patients with presumed benign leiomyoma, minimally invasive surgery with morcellation may be considered; however, when uterine sarcoma is suspected, intact specimen removal and avoidance of tumour fragmentation are oncologically preferable. Therefore, although CRP lacks disease specificity and cannot independently establish malignancy, an elevated preoperative CRP level may contribute to the overall suspicion of sarcoma when interpreted together with age, imaging features, and clinical presentation. In this context, CRP may help support a more cautious surgical strategy.

Importantly, CRP should not be interpreted as a standalone diagnostic tool but rather as part of a multimodal assessment framework that includes clinical evaluation and imaging findings. Future research should focus on integrating CRP with other emerging biomarkers, imaging criteria, and possibly machine-learning-based prediction models to improve diagnostic accuracy [37,38]. Additionally, prospective studies with larger cohorts are required to establish clinically relevant cut-off values and to assess diagnostic performance metrics such as sensitivity, specificity, and area under the receiver operating characteristic curve.

The biological mechanisms underlying elevated CRP levels in uterine sarcoma remain incompletely understood. It is likely that tumour-associated inflammatory responses, mediated by cytokines such as interleukin-6, contribute to hepatic CRP production. This systemic inflammatory response may reflect tumour burden or aggressive tumour behaviour, although our study did not demonstrate a clear association between CRP levels and tumour aggressiveness. Further translational studies are needed to elucidate these mechanisms.

Several limitations of this study should be acknowledged. First, this was a retrospective single-centre study with a relatively small sample size, which reflects the rarity of uterine sarcomas but may limit statistical power and generalizability. Second, the control group was substantially younger than the sarcoma group, and age may influence systemic inflammatory parameters. Third, CRP is a non-specific inflammatory marker and may be affected by confounding factors, including obesity, smoking, metabolic disease, and other subclinical inflammatory states. Fourth, several potentially relevant confounders were not included in the analysis. Although leukocyte count, BMI, and information on metabolic comorbidities were available for at least part of the cohort, these variables were not systematically analyzed in the present study. In addition, differential blood count was not routinely performed preoperatively at our institution, and smoking status was not consistently documented. These factors may have influenced systemic inflammatory parameters and should be considered in future studies. Fifth, postoperative CRP values may have been influenced by operative factors such as surgical approach, tissue trauma, and timing of blood sampling [39]. Although surgical approach data were available, the present study was not designed to compare inflammatory responses between operative techniques, and this potential confounder was not formally analyzed. Sixth, tumour size, detailed anatomical characteristics, and immunohistochemical profiles were not uniformly retrievable and were not analyzed in correlation with CRP. Finally, although receiver operating characteristic analysis was performed as an exploratory assessment, the limited sample size and retrospective design restrict the robustness of diagnostic cut-off estimation and performance metrics. Therefore, the diagnostic value of CRP should be validated in larger prospective cohorts.

Despite these limitations, the present study provides novel evidence suggesting that preoperative CRP levels may serve as a simple, inexpensive, and widely available adjunct marker in the diagnostic evaluation of suspected uterine sarcoma. Future studies involving larger, multicentre cohorts and prospective designs are needed to validate these findings and to determine whether CRP could be integrated into multimodal diagnostic algorithms for preoperative risk stratification.

## 5. Conclusions

Uterine sarcomas are rare but highly aggressive malignancies that remain difficult to distinguish from benign leiomyomas before surgery. Accurate preoperative differentiation is essential for appropriate surgical planning and to minimize the risk of tumour dissemination.

The results of this study demonstrate that patients with uterine sarcoma exhibit significantly higher preoperative serum CRP levels compared with patients with leiomyoma. These findings suggest that systemic inflammatory markers may contribute to preoperative risk assessment in patients presenting with uterine masses.

Given its wide availability, low cost, and routine use in clinical practice, CRP may represent a useful adjunct biomarker to support the preoperative evaluation of suspected uterine sarcoma. However, due to its non-specific nature, CRP should be interpreted within the broader clinical and radiological context.

Further prospective multicentre studies are required to validate the diagnostic value of CRP and to determine whether it could be incorporated into multimodal diagnostic algorithms for improving preoperative identification of uterine sarcomas.

## Figures and Tables

**Table 1:** Preoperative CRP serum values in patients with uterine leiomyoma and uterine sarcoma.

Group	Total (*n*)	Normal CRP (≤5 mg/L), *n* (%)	Elevated CRP (>5 mg/L), *n* (%)	Mean CRP (mg/L ± SD)
Leiomyoma	39	38 (97.4%)	1 (2.6%)	0.4 ± 1.6
Sarcoma (overall)	39	18 (46.2%)	21 (53.8%)	26.4 ± 46.8

CRP—C-reactive protein; SD—standard deviation.

**Table 2:** Distribution of sarcoma subtypes among patients with elevated preoprative CRP (>5 mg/L).

Sarcoma Subtype	*n* (%) Among Elevated CRP Cases
Leiomyosarcoma	7 (33.3%)
Adenosarcoma	6 (28.6%)
Undifferentiated sarcoma	5 (23.8%)
Low-grade ESS	1 (4.8%)
High-grade ESS	2 (9.5%)

ESS—endometrial stromal sarcoma. Percentages for sarcoma subtypes are calculated among patients with elevated CRP levels (*n* = 21), not among all sarcoma cases.

**Table 3:** Preoperative and postoperative CRP levels according to uterine sarcoma subtype.

Sarcoma Subtype	Preoperative CRP (mg/L ± SD)	Postoperative CRP (mg/L ± SD)	CRP Change (mg/L ± SD)
Undifferentiated sarcoma	94.4 ± 50.2	117.6 ± 62.1	23.2 ± 12.5
High-grade ESS	54.2 ± 40.3	76.8 ± 48.6	22.6 ± 15.7
Leiomyosarcoma	56.8 ± 41.9	72.3 ± 51.4	15.5 ± 10.3
Low-grade ESS	11.0 ± 8.5	15.7 ± 10.4	4.7 ± 2.8
Adenosarcoma	17.3 ± 9.6	25.1 ± 14.2	7.8 ± 4.6

CRP—C-reactive protein; SD—standard deviation; and ESS—endometrial stromal sarcoma.

**Table 4:** Preoperative and postoperative CRP levels according to tumour aggressiveness.

Tumour Aggressiveness	Preoperative CRP (mg/L ± SD)	Postoperative CRP (mg/L ± SD)	CRP Change (mg/L ± SD)
Aggressive	68.5 ± 45.3	89.2 ± 55.7	20.7 ± 14.1
Non-aggressive	14.2 ± 11.8	20.6 ± 13.9	6.4 ± 3.8

CRP—C-reactive protein; SD—standard deviation.

## Data Availability

The data presented in this study are available from the corresponding author upon reasonable request. The data are not publicly available due to ethical restrictions and patient privacy regulations related to the protection of sensitive personal health data.

## Abbreviations

The following abbreviations are used in this manuscript:

AUC	Area under the curve
CRP	C-reactive protein
ESS	Endometrial stromal sarcoma
IQR	Interquartile range
ROC	Receiver operating characteristic
SPSS	Statistical Package for the Social Sciences
WHO	World Health Organization
